# Left atrial function index predicts poor outcome in STEMI patients treated with percutaneous coronary intervention

**DOI:** 10.1038/s41598-023-33257-1

**Published:** 2023-06-21

**Authors:** Yi Tang, Pei Huang, Zhibin Liu, Yijin Tang, Wei Liu, Chang She, Changqing Zhong, Jianqiang Pei, Qinghua Fu, Liang Zhang, Yi Zhang

**Affiliations:** 1 Department of Cardiology, Hunan Provincial People’s Hospital, The First Affiliated Hospital of Hunan Normal University, Clinical Medicine Research Center of Heart Failure of Hunan Province, Hunan Normal University, Changsha, 410005 China; 2 Department of Cardiology, Changsha Hospital of Traditional Chinese Medicine (Changsha Eighth Hospital), Changsha, 410199 Hunan China

**Keywords:** Cardiology, Diseases, Medical research

## Abstract

The prognostic value of the left atrial function index (LAFI) in acute ST segment elevation myocardial infarction (STEMI) patients treated with percutaneous coronary intervention (PCI) is unknown. This study sought to determine whether the LAFI predicts prognosis in STEMI patients treated with PCI. Patients with newly diagnosed STEMI who were treated with PCI in Hunan Provincial People's Hospital from March 2020 to October 2020 were prospectively enrolled. All patients underwent transthoracic echocardiography at baseline and follow-up. The endpoint events included rehospitalization due to unstable angina, nonfatal myocardial infarction, rehospitalization due to heart failure and cardiovascular death. A total of 156 STEMI patients treated with PCI were studied with a median follow-up of 14 months. Forty-eight patients had endpoint events. The LAFI had the highest area under the receiver operating characteristic curve (AUC) predicting the endpoint events, with an AUC of 0.90 (95% CI 0.84–0.94). Multivariate Cox analysis demonstrated that only the LAFI (HR: 0.91, 95% CI 0.87–0.96, *P* < 0.0001) was independently predictive of endpoint events. Kaplan‒Meier survival curves showed that patients with an LAFI ≤ 42.25 cm/cc/m^2^ had more events than patients with an LAFI > 42.25 cm/cc/m^2^ (HR: 19.15, 95% CI 8.90–41.21, *P* < 0.001). The LAFI is a strong and independent predictor of events in STEMI patients treated with PCI.

## Introduction

Acute ST segment elevation myocardial infarction (STEMI) is the most acute manifestation of coronary artery disease. Studies have found a decrease in mortality in patients with STEMI in parallel with greater use of primary percutaneous coronary intervention (PCI)^1^. However, some patients will still experience adverse events, such as unstable angina, nonfatal myocardial infarction, heart failure after myocardial infarction and death even if they are treated with PCI^2^. It is very important to identify acute myocardial infarction patients with a higher risk of adverse events after myocardial infarction; therefore, we treat these patients with intensive drugs at the early stage.

Complete thrombotic occlusion in coronary vessels leads to myocardial necrosis and remodelling, which usually can cause left ventricular systolic and diastolic dysfunction. The left ventricular ejection fraction (LVEF) obtained from echocardiography is usually used to assess left ventricular (LV) systolic dysfunction, which can predict a poor outcome in patients with acute myocardial infarction^3^. Left atrial (LA) volume is a marker of the severity and duration of left ventricular diastolic dysfunction, which can also predict the prognosis of patients with acute myocardial infarction^4,5^.

The left atrial function index (LAFI) is a ratio that incorporates analogues of cardiac output, atrial reservoir function and LA size, which reflects LV systolic and diastolic function, as well as LA function^6,7^. The LAFI has been proven to be a good predictor of hospitalization for HF in patients with preserved ejection fraction and coronary heart disease^8^ and could also predict long-term survival in stable outpatients with systolic heart failure^6^. However, whether the LAFI could be used to predict the outcome of patients with STEMI treated with PCI is unknown. This study was designed to explore the value of the LAFI in the prognostic evaluation of patients with STEMI treated with PCI.

## Methods

### Study population

Patients who were diagnosed with acute STEMI and received emergent percutaneous coronary intervention (PCI) in Hunan Provincial People's Hospital between March 2020 and October 2020 were enrolled. The diagnostic criteria for STEMI were based on clinical guidelines^1^. Patients with poor imaging of the atrium and moderate to severe degrees of mitral regurgitation were excluded. This research was conducted in compliance with the Declaration of Helsinki and was approved by the Ethics Committee of Hunan Provincial People's Hospital (2018-50). Informed consent was obtained from all enrolled patients.

A total of 173 patients with STEMI who underwent PCI were screened. Six patients (3.5%) with poor imaging of the atrium, two patients (1.2%) with moderate to severe mitral regurgitation and nine patients (5.2%) lost to follow-up were excluded, and the remaining 156 patients (90.1%) were finally included in the analysis of our study. Among the 17 patients who were not included in the analysis, 5 patients were Killip class I,8 patients were Killip class II, 3 patients were Killip class III, 1 patient was Killip class IV.

### Echocardiographic methods

We performed resting transthoracic echocardiography (GE vivid E9, America) for all patients within 2 days after they underwent emergent PCI, and 98.1% of the patients underwent transthoracic echocardiography within 6 h. Transthoracic echocardiography was performed in the standard left lateral recumbent and supine positions. Routine M-mode and 2-dimensional echocardiography were performed using a standard protocol^9^. The left atrial end-diastolic volume (LAEDV) and left atrial end-systolic volume (LAESV) were determined by averaging LAEDV and LAESV measurements from the apical two- and four-chamber views using the recommended Simpson’s biplane summation of disks method. The LA emptying fraction (LAEF) was calculated as ([LAESV – LAEDV]/LAESV) × 100. The left atrial end systolic volume index (LAESVi) was calculated by dividing LAESV by the body surface area (BSA). LV end-diastolic and end-systolic volumes were measured using Simpson’s method in the apical-4 chamber and the apical-2 chamber view. Stroke volume was calculated as (LV end-diastolic volume − LV end-systolic volume), and LV ejection fraction was calculated as (Stroke volume/LV end-diastolic volume) × 100. The left ventricular outflow tract velocity time integral (LVOT-VTI) was measured by manually tracing pulsed Doppler velocities in the left ventricular outflow tract in apical 5-chamber views. The final measures were derived by averaging the measurements over ≥ 3 cardiac cycles. The LAFI was calculated using a previously validated formula: LAFI = (LAEF × LVOT-VTI)/LAESVi^7^. Stevenson formula was used to calculate BSA (BSA (m^2^) = 0.0061 × Height (cm) + 0.0128 × Weight (kg) − 0.1529).

### Clinical assessment and follow-up

Basic demographic data, biochemical tests, and Killip classifications were collected at baseline after the patients underwent emergent PCI. The N-terminal fragment of pro B-type natriuretic peptide (NT-proBNP) was also determined at baseline after the patients underwent emergent PCI using the chemiluminescence immunoassay method (Wantaikairui, XiaMen, China) in the Department of Laboratory Medicine of Hunan Provincial People’s Hospital.

All enrolled patients were followed up telephonically at 1, 3, 6, 12, and 18 months after discharge, and the endpoint events during this period were recorded. The endpoint events were defined as rehospitalization due to unstable angina, nonfatal myocardial infarction, rehospitalization due to heart failure and cardiovascular death. The follow-up period was completed on September 20, 2021.

### Statistical methods

Continuous variables with a normal distribution are expressed as the means ± standard deviation ($\overline{x} \pm s$), and continuous variables with a nonnormal distribution are represented by the medians and quartiles (IQRs). One-way ANOVA, Student’s *t-test* or the Mann‒Whitney U test was used for comparisons as appropriate. Categorical variables are expressed as n (%), and the chi-square (χ^2^) test was used for categorical variables. Pearson’s or Spearman’s correlation coefficient was used for bivariate correlation analysis. Receiver operating characteristic (ROC) curves were used to judge the performance of variables in prognostic prediction and to determine the best cut-off point.

Univariate and multivariate Cox proportional hazards models and Kaplan‒Meier curves were used for survival analysis. Some important clinical variables and other variables with statistically significant differences between the two groups (P < 0.05) were included in the univariate Cox regression model. The variables predictive of events by univariate analysis (P < 0.05) were then entered into a multivariable Cox regression. Since there were not enough events, to avoid overfitting, we included white blood cell count (WBC), NT-proBNP, type 2 diabetes mellitus (T2DM), Killip classification, multivessel coronary artery disease (MVD), LAFI, LVEF, and LVEDV in a multivariable Cox regression. A two-tailed *P* value < 0.05 was considered statistically significant. The ROC curve was analysed using MedCalc v19.3.0, and the remaining assays were analysed using SPSS 23.0.

## Results

### Baseline characteristics

The mean age was 63.39 ± 11.36 years, and 75.7% of patients were men. The median follow-up time was 13.95 ± 4.05 months, and 48 patients developed events during the follow-up period, including 10 patients readmitted due to unstable angina pectoris, 26 patients readmitted due to heart failure, 5 patients with nonfatal myocardial infarction, and 7 patients with cardiovascular death.

### Differences in variables between groups

Patients were divided into two groups based on whether they experienced events. Patients with events (Group 1) had a higher proportion of T2DM and multivessel coronary artery disease, a poorer Killip classification, and higher NT-proBNP and WBC than patients without events (Group 2). In addition, patients who presented with events had significantly lower LAEF, LAFI, LVEF, and LVOT-VTI and higher LAESVi and LVEDV (Table 1).

**Table 1:** Comparison of baseline data between patients with or without events.

Clinical characteristics	Patients with events (n = 48)	Patients without events (n = 108)	P value
Male, n (%)	36 (75.0%)	88 (74.0%)	NS
Age, y	64.35 ± 11.35	62.42 ± 11.37	NS
BMI, kg/m^2^	24.42 ± 3.79	23.66 ± 3.26	NS
Smoking, n (%)	31 (64.5%)	82 (75.9%)	NS
Hypertension, n (%)	28 (58.8%)	62 (57.4%)	NS
Atrial fibrillation	2 (4.16)	1 (0.92)	NS
T2DM, n (%)	23 (47.9%)	20 (18.5%)	0.001
Previous CI, n (%)	4 (8.3%)	5 (4.6%)	NS
Previous MI, n (%)	8 (16.6%)	10 (9.2%)	NS
The Killip classification, n (%)			0.001
I/II	40 (83.3%)	103 (95.4%)	
III/IV	8 (16.7%)	5 (4.6%)	
Biochemical parameters			
WBC, × 10^9^/L	11.41 ± 4.03	9.30 ± 2.61	0.001
TC, mmol/l	4.63 ± 1.28	4.62 ± 1.15	NS
LDL, mmol/l	2.79 ± 0.91	2.84 ± 0.97	NS
eGFR, ml/min/1.73m^2^	83.66 ± 41.15	97.75 ± 107.96	NS
TB, µmol/l	13.89 ± 6.855	14.14 ± 6.37	NS
NT-proBNP, ng/L	5949.18 ± 7974.56	2070.53 ± 3846.44	< 0.001
Coronary arteriography			
Culprit vessel, n (%)			NS
LAD	30 (62.5%)	58 (53.8%)	
RCA	14 (29.2%)	41 (37.9%)	
LCX	4 (8.3%)	9 (8.3%)	
TIMI flow before PCI, n (%)			NS
TIMI 0	29 (60.4%)	51 (47.3%)	
TIMI 1	6 (12.5%)	9 (8.3%)	
TIMI 2	5 (10.4%)	15 (13.8%)	
TIMI 3	8 (16.7%)	33 (30.6%)	
MVD, n (%)	46 (95.8%)	75 (69.4%)	0.001
Echocardiography			
LAESVi, mL/m^2^	28.98 ± 6.17	24.27 ± 5.12	< 0.001
LAEF, %	47.90 ± 9.92	58.15 ± 6.71	< 0.001
LVOT-VTI, cm	18.24 ± 3.39	21.73 ± 3.53	< 0.001
LAFI, cm/cc/m^2^	33.04 ± 11.33	53.80 ± 12.34	< 0.001
LVEF, %	41.95 ± 9.30	54.10 ± 8.91	< 0.001
LVEDV, mL	80.79 ± 18.10	66.49 ± 12.79	< 0.001
Therapeutics			
Aspirin, n (%)	47 (97.9%)	107 (99.0%)	NS
P2Y12 inhibitor, n (%)	48 (100.0%)	108 (100.0%)	NS
β-blocker, n (%)	42 (87.5%)	99 (91.6%)	NS
ACEI/ARB, n (%)	44 (91.6%)	96 (88.8%)	NS
Statin, n (%)	48 (100.0%)	107 (99.0%)	NS

ACEI/ARB angiotensin-converting enzyme inhibit or angiotensin receptor blocker; BMI: body mass index; CI: cerebral infarction; eGFR: estimated glomerular filtration rate; LAD: left anterior descending; LAEF: left atrial emptying index; LAESVi: left atrial end-systolic volume index; LAFI: left atrial function index; LCX: left circumflex artery; LDL: low density lipoprotein; LVEDV: left ventricular end-diastolic volume; LVEF: left ventricular ejection fraction; LVESV: left ventricular early diastolic volume; LVOT-VTI: left ventricular outflow tract velocity time integral; MI: myocardial infarction; MVD: multivessel coronary artery disease; NT-proBNP, NT-terminal B-type brain natriuretic peptide precursor; RCA: right coronary artery; T2DM: type 2 diabetes mellitus; TB: total bilirubin; TC: total cholesterol; WBC: white blood cell count.

### Correlation analysis of the LAFI

**Figure 1:**
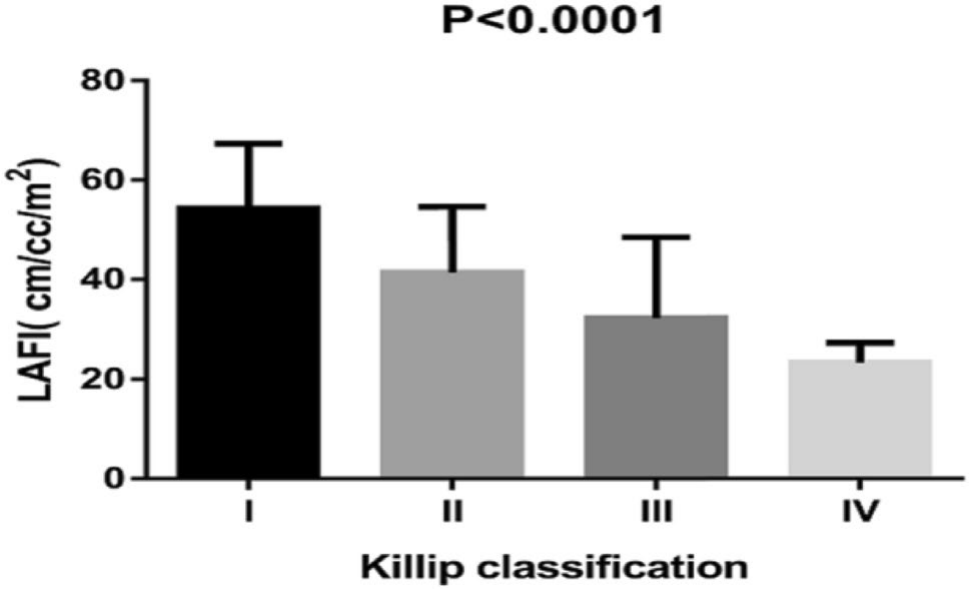
LAFI according to the Killip classification.

As the Killip classification increased, LAFI levels became significantly lower (I: 54.32 ± 13.04 cm/cc/m^2^; II: 41.41 ± 13.22 cm/cc/m^2^; III: 32.34 ± 16.18 cm/cc/m^2^; IV: 23.38 ± 3.96 cm/cc/m^2^) (Fig. 1). Compared to patients without atrial fibrillation (153 patients), LAFI levels were lower in patients with atrial fibrillation (3 patients) (38.99 ± 20.35 vs. 47.57 ± 15.30 cm/cc/m^2^), but the difference was not statistically significant (*P* > 0.05). LAFI levels correlated positively with LVEF and eGFR and negatively with age, NT-proBNP, WBC, and LVEDV (Table 2).

**Table 2:** Correlation analysis of the LAFI with variables.

Variables	r	P
Age	− 0.30	0.002
BMI	− 0.01	0.921
NT-proBNP	− 0.50	< 0.001
WBC	− 0.20	0.014
eGFR	0.22	0.006
LVEF	0.74	< 0.001
LVEDV	− 0.54	< 0.001

BMI: body mass index; eGFR: estimated glomerular filtration rate; LVEDV: left ventricular end-diastolic volume; LVEF: left ventricular ejection fraction; NT-proBNP: NT-terminal B-type brain natriuretic peptide precursor; WBC: white blood cell count.

### LAFI and the events

Univariate Cox regression analysis revealed that T2DM, WBC, NT-proBNP, MVD, Killip classification, and variables obtained from echocardiography were significant predictors of events (Table 3).

**Table 3:** Univariate Cox regression analysis.

Variables	χ^2^	HR (95%CI)	P value
Age	0.84	1.01 (0.99, 1.04)	0.35
Sex	0.01	0.96 (0.50, 1.85)	0.91
T2DM	13.86	2.94 (1.67, 5.18)	< 0.001
WBC	17.65	1.20 (1.10, 1.29)	< 0.001
eGFR	0.90	1.00 (0.99, 1.01)	0.342
NT-proBNP	25.65	1.00 (1.00, 1.00)	< 0.001
MVD	8.26	7.98 (1.93, 32.88)	0.004
Killip classification	22.24	2.64 (1.76, 3.95)	< 0.001
LAESVi	31.54	1.13 (1.08, 1.18)	< 0.001
LAEF	61.59	0.92 (0.91, 0.94)	< 0.001
LAFI	70.64	0.90 (0.88, 0.92)	< 0.001
LVEF	54.28	0.90 (0.87, 0.92)	< 0.001
LVEDV	35.24	1.04 (1.03, 1.06)	< 0.001
LVOT-VTI	23.47	0.82 (0.76, 0.89)	< 0.001

eGFR: estimated glomerular filtration rate; LAEF: left atrial emptying index; LAESVi: left atrial end-systolic volume index; LAFI: left atrial function index; LVEDV: left ventricular end-diastolic volume; LVEF: left ventricular ejection fraction; LVOT-VTI: left ventricular outflow tract velocity time integral; MVD: multivessel coronary artery disease; NT-proBNP: NT-terminal B-type brain natriuretic peptide precursor; T2DM: type 2 diabetes mellitus; WBC: white blood cell count.

**Figure 2:**
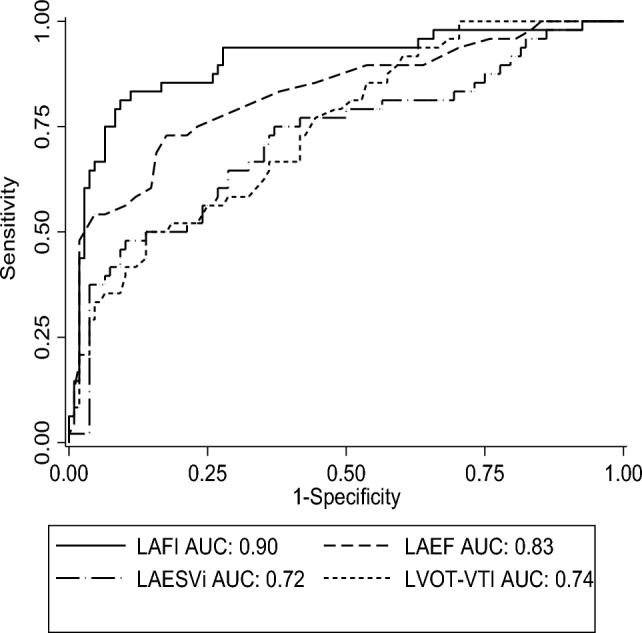
Receiver operating curve for endpoint events according to the LAFI and its components.

The discrimination of the LAFI for events was superior to each of its individual components (AUC, LAFI: 0.90, LAEF: 0.83, LVOT-VTI: 0.74, and LAESVi: 0.72) (Fig. 2). The LAFI components, LAESVi, LAEF, and LVOT-VTI, were all significant univariate predictors of events. However, the LAFI [hazard ratio (HR): 0.90, 95% confidence interval (CI) 0.84–0.97, *P* = 0.005] was the only significant predictor of events in multivariable Cox analysis, including the LAFI, LAESVi, LAEF, and LVOT-VTI.

Multivariable Cox analysis was also performed and included WBC, NT-proBNP, T2DM, Killip classification, MVD, LAFI, LVEF, and LVEDV. The results showed that the LAFI (HR: 0.91, 95% CI 0.87–0.96, *P* < 0.0001) was the only predictor of events (Table 4).

**Table 4:** Multivariate Cox regression analysis.

Variables	HR [95% CI]	P value
NT-proBNP	1.00 (1.00, 1.00)	0.591
WBC	1.07 (0.98, 1.16)	0.119
Killip classification	0.92 (0.51, 1.66)	0.776
T2DM	1.64 (0.90, 2.98)	0.104
MVD	1.45 (0.31, 6.74)	0.637
LAFI	0.91 (0.87, 0.96)	< 0.001
LVEDV	1.01 (0.99, 1.03)	0.478
LVEF	0.99 (0.95, 1.04)	0.776

NT-proBNP: NT-terminal B-type brain natriuretic peptide precursor; WBC: white blood cell count; T2DM: type 2 diabetes mellitus; MVD: multivessel coronary artery disease; LAFI: left atrial function index; LVEDV: left ventricular end-diastolic volume; LVEF: left ventricular ejection fraction.

The specificity and sensitivity for predicting the events and the area under the receiver operator characteristic curve (AUC) for each variable are shown in Table 5. Compared with other variables, the LAFI had the highest area under the receiver operating characteristic curve (AUC) predicting the events. The calculated optimal point of LAFI for predicting the events was ≤ 42.25 cm/cc/m^2^, with a sensitivity and specificity of 88.30 and 88.90%, respectively.

**Table 5:** ROC curve analysis for variables in predicting events.

Variables	AUC (95%CI)	Sensitivity/specificity	P value
LAFI	0.90 (0.84, 0.96)	83.30%/88.90%	< 0.001
LVEF	0.83 (0.75, 0.90)	72.90%/82.40%	< 0.001
NT-proBNP	0.76 (0.68, 0.85)	66.70%/79.60%	< 0.001
LVEDV	0.75 (0.67, 0.84)	62.50%/83.30%	< 0.001
Killip Classification	0.70 (0.61, 0.79)	72.90%/64.80%	< 0.001
WBC	0.66 (0.57, 0.75)	50.00%/75.90%	0.001
T2DM	0.65 (0.55, 0.75)	47.9%/81.5%	0.003
MVD	0.63 (0.54, 0.72)	95.8%/30.6%	0.009

LAFI: left atrial function index; LVEDV: left ventricular end-diastolic volume; LVEF: left ventricular ejection fraction; MVD: multivessel coronary artery disease; NT-proBNP, NT-terminal B-type brain natriuretic peptide precursor; T2DM: type 2 diabetes mellitus; WBC: white blood cell count.

**Figure 3:**
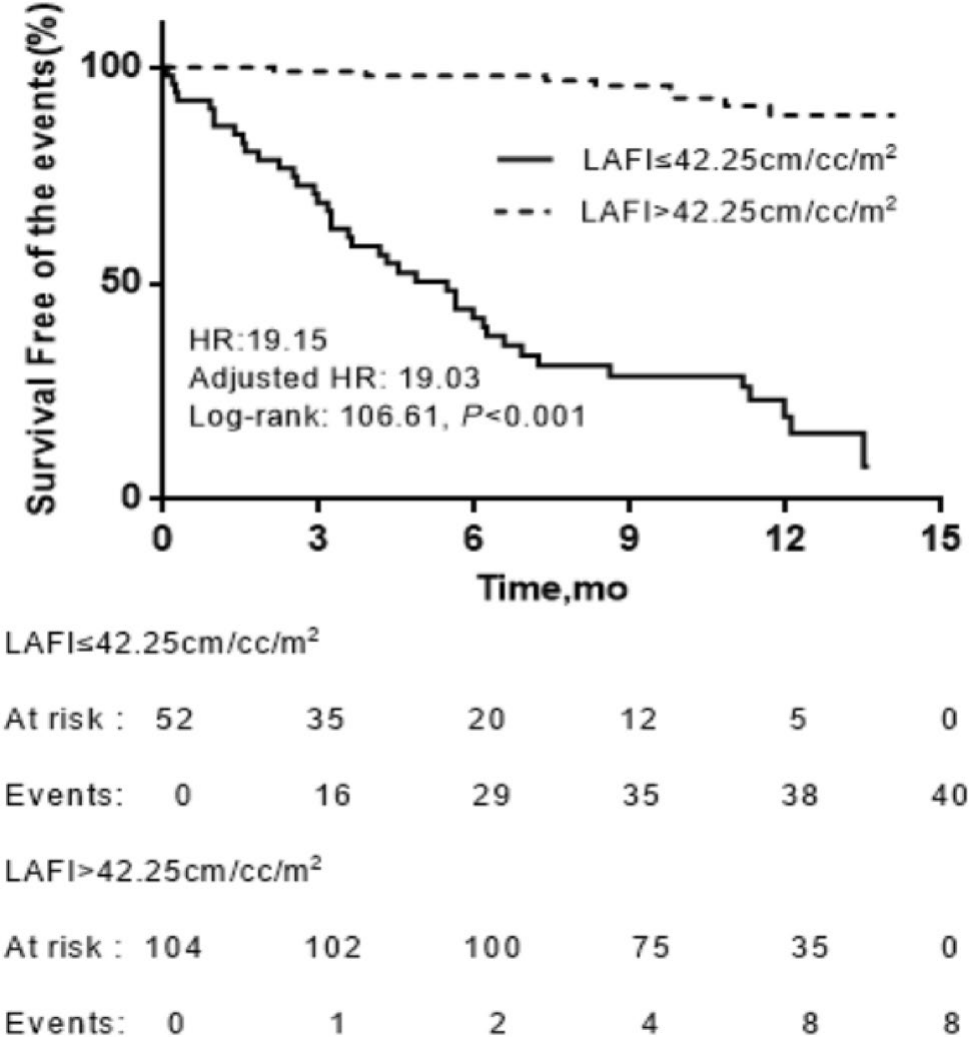
Kaplan–Meier analysis of LAFI for events. Adjusted HR indicates hazard ratio (HR) adjusted for age.

Patients with an LAFI ≤ 42.25 cm/cc/m^2^ had a worse survival rate than patients with an LAFI > 42.25 cm/cc/m^2^. The unadjusted HR was 19.15 (95% CI 8.90–41.21), and after adjustment for age, the HR was 19.03 (95% CI 8.83–41.01). The Kaplan–Meier curves of the LAFI are shown in Fig. 3.

## Discussion

In this study, we first evaluated the prognostic value of the LAFI in patients with STEMI treated with PCI. The results showed that the LAFI was negatively correlated with NT-proBNP, and its levels were significantly decreased as the Killip classification increased. In addition, patients with a lower LAFI were associated with poor prognosis, including rehospitalization due to unstable angina, nonfatal myocardial infarction, rehospitalization due to heart failure and death. Importantly, the prognostic value of the LAFI was independent of a wide range of clinical risk factors and laboratory and echocardiographic parameters.

Patients with acute myocardial infarction usually have left ventricular (LV) systolic and diastolic dysfunction. The LAFI combines stroke volume (LVOT-VTI), left atrium reservoir function (LAEF) and adjusted LA volume (LAESVi). It increases proportionally to left atrium reservoir function and stroke volume but is inversely proportional to left atrium volume^7^. The LAFI not only reflects LA function but also reflects both LV systolic and diastolic function^6^. In addition, the LAFI can easily be performed by an experienced operator, and its calculation does not require any additional echocardiographic views^10^. Thus, the combination of LA function and LV systolic and diastolic functions in one index may provide greater prognostic information in patients with acute myocardial infarction.

In our study, the LAFI was associated with the Killip classification, NT-proBNP, and LVEF in patients with STEMI treated with PCI. Furthermore, LAFI could independently predict the events after adjusting for significant confounders, and the results supported that the LAFI was useful in risk stratification to identify patients with STEMI treated with PCI who are at high risk for adverse events. The results of our study were also consistent with the results of studies conducted in patients with preserved ejection fraction and coronary heart disease and patients with stable systolic heart failure^6,8^. Welles et al. demonstrated that the LAFI was a good predictor of heart failure hospitalization in patients with preserved ejection fraction and coronary heart disease after a median follow-up of 7.9 years^8^. Sargento et al. found that the LAFI could also predict long-term survival in 203 stable systolic heart failure outpatients with a left ventricular ejection fraction < 40%^6^. However, the calculated optimal point of the LAFI for predicting the events in our patients with STEMI was higher than that in patients with a left ventricular ejection fraction < 40% (42.25 vs. 16.57 cm/cc/m^2^). This may be attributed to different study groups, as our study enrolled all patients with STEMI treated with PCI, regardless of the left ventricular ejection fraction.

Atrial fibrillation can affect LA contractile function and decrease the LAFI; the LAFI is lower in subjects with atrial fibrillation than in subjects without atrial fibrillation^11^, which was shown in our study. However, because the sample of patients with atrial fibrillation was relatively small, the difference was not statistically significant.

## Study limitations

There are several limitations to our study. First, we did not measure the LAFI before patients underwent PCI; due to the emergent condition, it was important to open the occluded vessels when the patients had STEMI. Second, our study was a single-centre study, and the sample size was relatively small. Third, the CIs for survival in the multivariate analysis were quite wide, which reduces the power of their analysis. A multicentre study with a large sample size will be required to further validate these results.

## Conclusions

The LAFI is a strong and independent predictor of events in patients with STEMI treated with PCI. The LAFI may be useful for risk stratification in patients with STEMI treated with PCI and identifying patients at high risk of events (Supplementary Information).

## Supplementary Information

Supplementary Information.

## Data Availability

The datasets used and/or analysed during the current study available from the corresponding author on reasonable request.
